# Invention of Hollow Zirconium Tungesto-Vanadate at Nanotube Morphological Structure for Radionuclides and Heavy Metal Pollutants Decontamination from Aqueous Solutions

**DOI:** 10.1186/s11671-015-1180-0

**Published:** 2015-12-09

**Authors:** M. F. Elkady, H. Shokry Hassan

**Affiliations:** Fabrication Technology Department, Advanced Technology and New Materials Research Institute (ATNMRI), City of Scientific Research and Technology Applications (SRTA City), Alexandria, Egypt; Chemical and Petrochemical Engineering Department, Egypt-Japan University of Science and Technology (E-Just), New Borg El-Arab City, Alexandria Egypt; Electronic Materials Researches Department, Advanced Technology and New Materials and Research Institute (ATNMRI), City of Scientific Research and Technological Applications, New Borg El-Arab City, Alexandria 2193 Egypt

**Keywords:** Nanotube, Zirconium tungesto-vanadate, Microwave technology, Cation exchanger, Strontium ions

## Abstract

Zirconium tungesto-vanadate cation exchange material was successfully architectured at open ended nanotubes morphological structure in the presence of polyvinyl alcohol as a stabilizing agent using microwave route. The ion exchange capacity (IEC) of the material was recorded as 4.8 meq/g of about 640 m^2^/g for a specific surface area. The x-ray diffraction pattern of the material implies its crystallinity. Both scanning and transmission electron microscopes identified the average aspect ratio of the architectured nanotubes as 6.5 and its hollow structure. The material posed 96.4 % cadmium ion decontamination within 90 min compared with 84 % strontium decontamination at the same time.

## Background

Water scarcity represents one of the problems that the world is facing now, where most of the countries are suffering from acute shortages of water for the degree of drought. Saving of huge amounts from industrial wastewater that discharged to the external environment may be participated in solving two problems in parallel, which are the water scarcity and water pollution. So, most of environmental researches have been focused on industrial wastewater treatment and the possibility of its reuse for the industrial purposes in order to save water consumption and decrease water pollution [1]. However, in order to recycle the treated industrial wastewater or even discharge clean wastewater to the external environment, the industrial wastewater should be completely free from the heavy metals to avoid their enormous risks on the vital life or even on the industrial machining system. Among the various heavy metals, cadmium ion is a well-known toxic metal, considered as a priority pollutant, and its adverse effects are well documented [2]. On the other hand, nuclear energy is getting more and more widely used around the world nowadays as a clean and promising energy source [3].

Radioactive wastes generated from the nuclear power plant therefore become an environmental concern. In respect to the hazard of the radioactive waste to the environment and public health, this waste needs to be treated before being discharged to the environment [4]. In the low-level radioactive liquid waste (LLRLW) discharged from a nuclear power plant, the majority of nucleotides are fission products. These fission products can be categorized into three different groups: long-lived members such as ^87^Sr and ^133^Cs; medium-lived members such as ^91^Zr, ^93^Nb, ^140^Ce, ^141^Pr, ^101^Ru, ^103^Rh, ^145^Pm, and ^150^Sm; and short-lived nuclides with half-lives ranging from a few seconds to days that can be neglected [4]. Accordingly, both cesium and strontium radionuclides represent the main pollutants due to their large active periods. In this respect, it is of particular significance to propose highly efficient technique to trap both lead and strontium ions from the contaminated wastewaters.

Among the techniques of water treatment, the ion exchange separation technique is characterized over the other separation techniques by its simplicity and because it is cheap and not energy consumable. Inorganic cation exchange materials are characterized by their chemical, thermal, and radiation stabilities compared with the organic counterpart cation exchangers [5].

Zirconium-based ion exchangers have received attention because of their excellent ion exchange behavior and some important chemical applications in the field of ion exchanger, ion exchange membrane, and solid state electrochemistry. Accordingly, various hetero-polyacid salts based on zirconium (IV) have been reported in the literature as cation exchange materials. Among these hetero-polyacid salts, zirconium tungesto-vanadate nanoparticles as novel material establish its effectiveness for cationic ions decontamination [6]. This novel material has been studied for its synthesis, ion exchange behavior, and analytical applications. This novel material explored higher ion exchange capacity and higher stability at elevated temperature compared with the other previously prepared zirconium-based hetero-polyacid cation exchange material such as zirconium tungstatephenolate, zirconium tungstophosphate, zirconium arsenovanadate, and zirconium iodovanadate [7]. Preparations of both poorly crystalline and amorphous nano-zirconium tungesto-vanadate materials in spherical particles morphological structures have been reported earlier [6]. However, the production of crystalline zirconium tungesto-vanadate material with other morphological structure rather than the spherical nanoparticles was not investigated at the literature.

In this concern, this cation exchange material will be architectured in hollow nanotube morphological structure with crystalline structure to improve its surface area that by its role enhance its sorption efficiency for the pollutants. The cation exchange performance of fabricated nanotube zirconium tungesto-vanadate will be examined toward both the cadmium and strontium ions decontamination from polluted wastewaters.

## Experimental

### Synthesis of Nano-Zirconium Tungesto-Vanadate using Microwave Technique

**Fig. 1:**
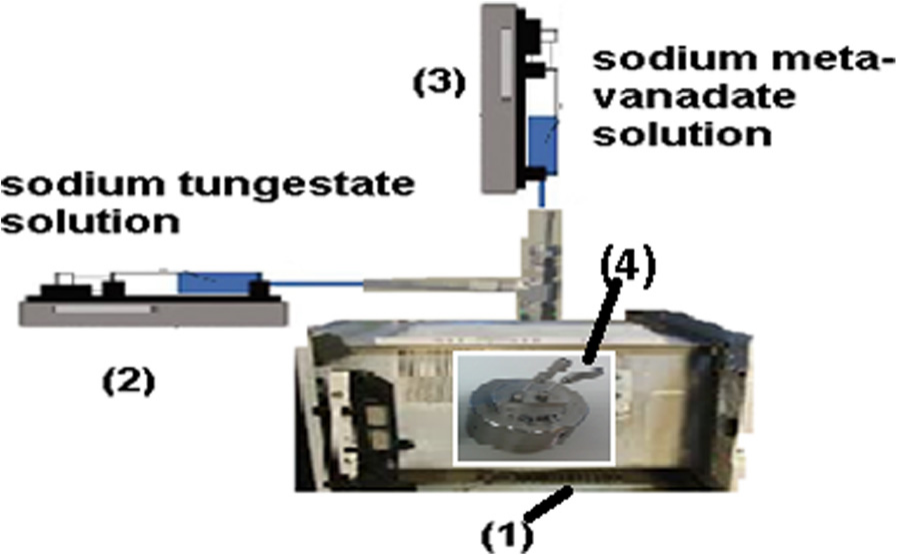
Microwave setup for hollow nanotube zirconium tungesto-vanadate production (1- microwave oven, 2- syringe pump for feeding sodium tungestate solution, 3- syringe pump for feeding sodium meta-vanadate solution, 4- stainless steel reactor)

Zirconium oxy chloride solution (1 M, 150 ml) was acidified with 0.05 M HCl and mixed with 0.1 M polyvinyl alcohol solution (polyvinyl alcohol (PVA) with Mwt = 72,000) at closed stainless steel reactor contains two upper slots, one is opened and the other closed as investigated in Fig. 1. This closed reactor was added into microwave oven that contains two upper stainless steel connections. The microwave power was adjusted at 8 W to maintain the reaction mixture at 70 °C. Two different solutions from 75 ml ammonium metavanadate (1 M) and 75 ml sodium tungstate (0.5 M) was added dropwise from the microwave connections simultaneously as indicated in Fig. 1. The solutions feeding occurred using two syringe pumps at constant flow rates of 75 ml/h. After completeness of the additional process, the reaction mixture was aged into the microwave oven until all the reaction solution evaporates and attains yellowish green dry powder material. The fine-resulted powder material was immersed in 1 M nitric acid for 1 day to be transformed to its hydrogen form and neutralize any PVA-stabilizing agent at the material structure (if present). The ion exchange capacity and the physical properties of the prepared material were determined.

### Characteristics of the Prepared Nano-Zirconium Tungesto-Vanadate

The physicochemical properties of prepared zirconium tungesto-vanadate material were examined using ion exchange capacity (IEC), x-ray diffraction (XRD), Brunauer–Emmett–Teller for surface area (BET), scanning electron microscope (SEM), and transmission electron microscope (TEM).

#### Ion Exchange Capacity

Acid-base titration process took place of the residual solution resulted from mixing of 0.5 g zirconium tungesto-vanadate in its H^+^ form with 50 ml of 1 M NaCl solution under vigorously stirring at room temperature overnight. The liberated hydrogen at the solution that is equivalent to the material ion exchange capacity was determined [5].

#### Surface Area (BET)

Based on the nitrogen chemisorption physisorption analyzer (Beckman Coulter AS3100, USA), the surface area of the prepared zirconium tungesto-vanadate was established after samples outgassed at 200 °C for 180 min.

#### X-Ray Diffraction

In order to determine the crystalline structure of the prepared zirconium tungesto-vanadate, the material was scanned at 4°min^−1^ for 2θ values between 10° and 80° using Schimadzu-7000 diffractometer with CuKα radiation beam (*λ* = 0.154060 nm).

#### Scanning Electron Microscope

The morphological structure of the prepared zirconium tungesto-vanadate was established after gold sputtering material and imaging using JEOL JSM 6360LA scanning electron microscope.

#### Transmission Electron Microscope

In order to confirm the material morphology, a small drop from ethanol-dispersed powder material was deposited on copper grid (pre-coated with a carbon film) and dried at air lab then examined using TEM (JEOL JEM-1230, Japan).

### Strontium and Cadmium Affinity of Nano-Zirconium Tungesto-Vanadate

The cadmium and strontium affinity of prepared zirconium tungesto-vanadate were compared using batch technique. A specific weight from prepared zirconium tungesto-vanadate (0.25g) was equililibrated with 100 ml solution from the synthetic waste solution contaminated with either cadmium or strontium ions with 50 ppm concentration under continuous shaking for different time intervals. The synthetic polluted solutions were prepared through dissolving appropriate amounts from either the cadmium chloride or strontium nitrate salts to prepare 1000 ppm from cadmium- and strontium-polluted solutions, respectively. These initial polluted solutions were diluted to 50 ppm to prepare the synthetic cadmium and strontium waste solutions. The reaming concentration of ions (cadmium and strontium) present at the solutions after the treatment process was measured using inductive coupled plasma mass spectrophotometer (ICP-AES).

## Results and Discussion

### Zirconium Tungesto-Vanadate Ion Exchange Capacity and Its Surface Area

It is well known that the improvement in the surface area of the ion exchange material augments its sorption efficiency for the pollutants present in contaminated water. Accordingly, the ion exchange capacity of zirconium tungesto-vanadate prepared at hollow nanotube morphological structure using microwave technique that recorded as 4.8 meq/g is much higher than its counterpart zirconium tungesto-vanadate material prepared at nanoparticle morphology using sol-gel technique, where the previous prepared recorded only 2.5 meq/g ion exchanges capacity value as stated at the literature [6]. This result may be returned to the high specific surface area of the fabricated hollow nanotube structure that measured as 640 m^2^/g compared with the previously prepared nanoparticle structure material. This comparable high surface area may be owed to the action of the PVA as a stabilizing agent during zirconium tungesto-vanadate formation at the microwave radiation that may force its orientation at the hollow nanotube morphological structure due to the high viscosity of the polymer solution and the vibration effect of the microwave electromagnetic waves [8]. The main effect of a stabilizing agent on a material particle size and its distribution may be due to the steric effect and chemical bonding between the long polymer chain and the synthesized zirconium tungesto-vanadate molecules [9]. The stabilization of colloidal zirconium tungesto-vanadate molecules with the long polymeric chains of PVA (Mwt = 72,000) in water is often discussed by the adsorption of the polymer on the colloidal material molecules. The interaction between the surface of the material molecules and the polymer chains is considered to be hydrophobic [10]. These large adsorbates provide a steric barrier which prevents close contact of formed zirconium tungesto-vanadate nanoclusters in hollow tube morphology to each other that maintains the nanotube morphology of produced zirconium tungesto-vanadate material. The hollow nanotubes clusters may be formed as a microwave action that forces the growth of adsorbed zirconium tungesto-vanadate molecules onto the polymer surface as a template to form the 3D nanotube structure [11].

### Zirconium Tungesto-Vanadate Crystalline Structure (XRD)

**Fig. 2:**
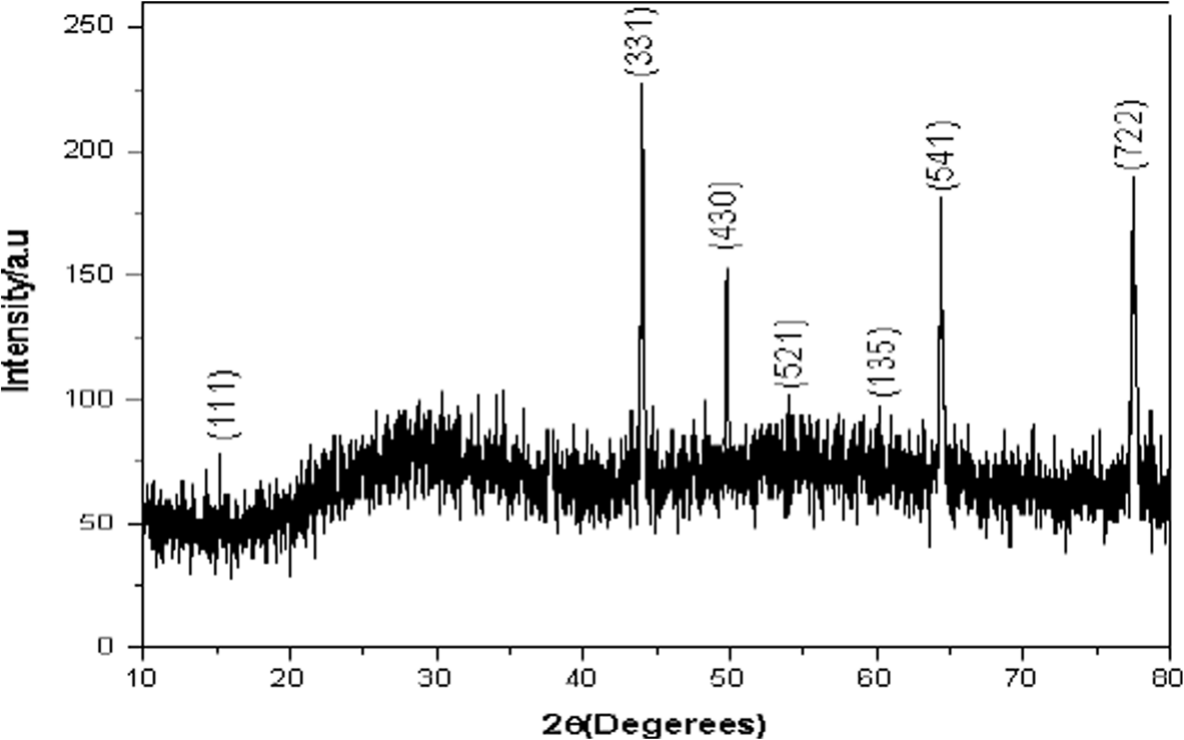
XRD of nanotube zirconium tungesto-vanadate cation exchange material

Figure 2 investigated the X-ray diffraction pattern of the prepared zirconium tungesto-vanadate and its orientation planes. The XRD patterns exhibited strong diffraction peaks at 43.1°, 51.6°, 65.97°, and 78.67° indicating well-defined zirconium tungesto-vanadate [6]. All peaks are in good agreement with the standard spectrum (JCPDS no.: 01-087-1528 and 01-088-0586). This result elucidated that the material is composed from zirconium tungsten oxide and zirconium vanadium oxide mixture with cubic crystal configurations. Moreover, as the action of the microwave beams, the material was produced at very pure crystalline structure [12] compared with the amorphous structure of the previously prepared zirconium tungesto-vanadate material that produced using sol-gel technique [6].

### Zirconium Tungesto-Vanadate Morphological Structure (SEM and TEM)

**Fig. 3:**
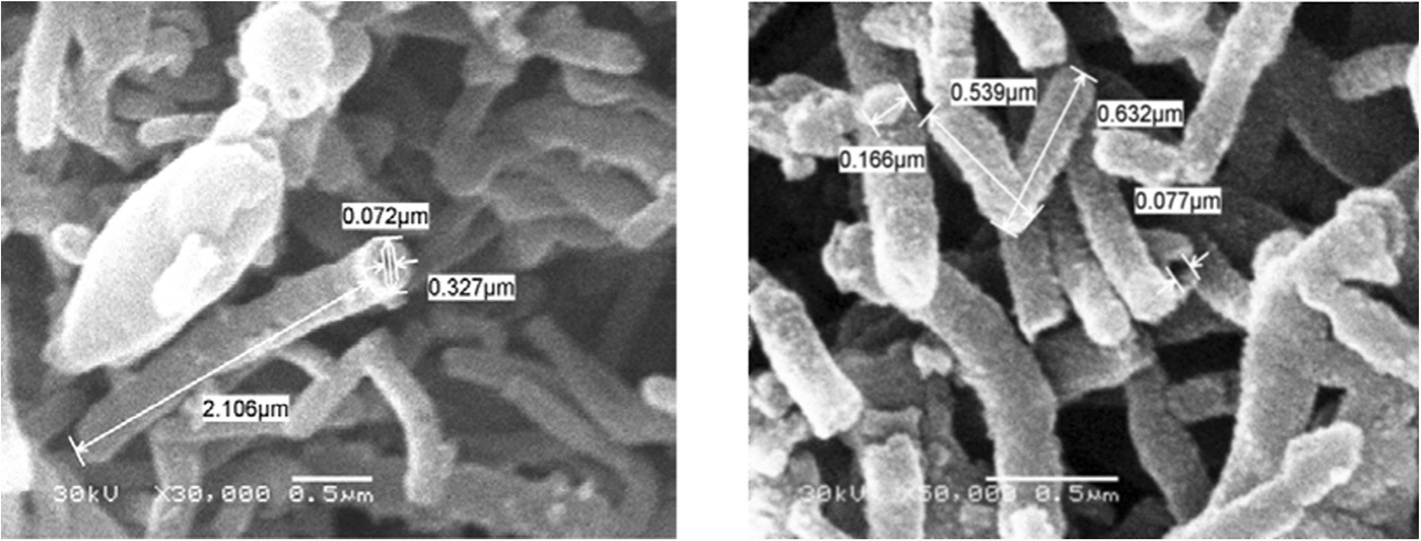
SEM of nanotube zirconium tungesto-vanadate cation exchange material

**Fig. 4:**
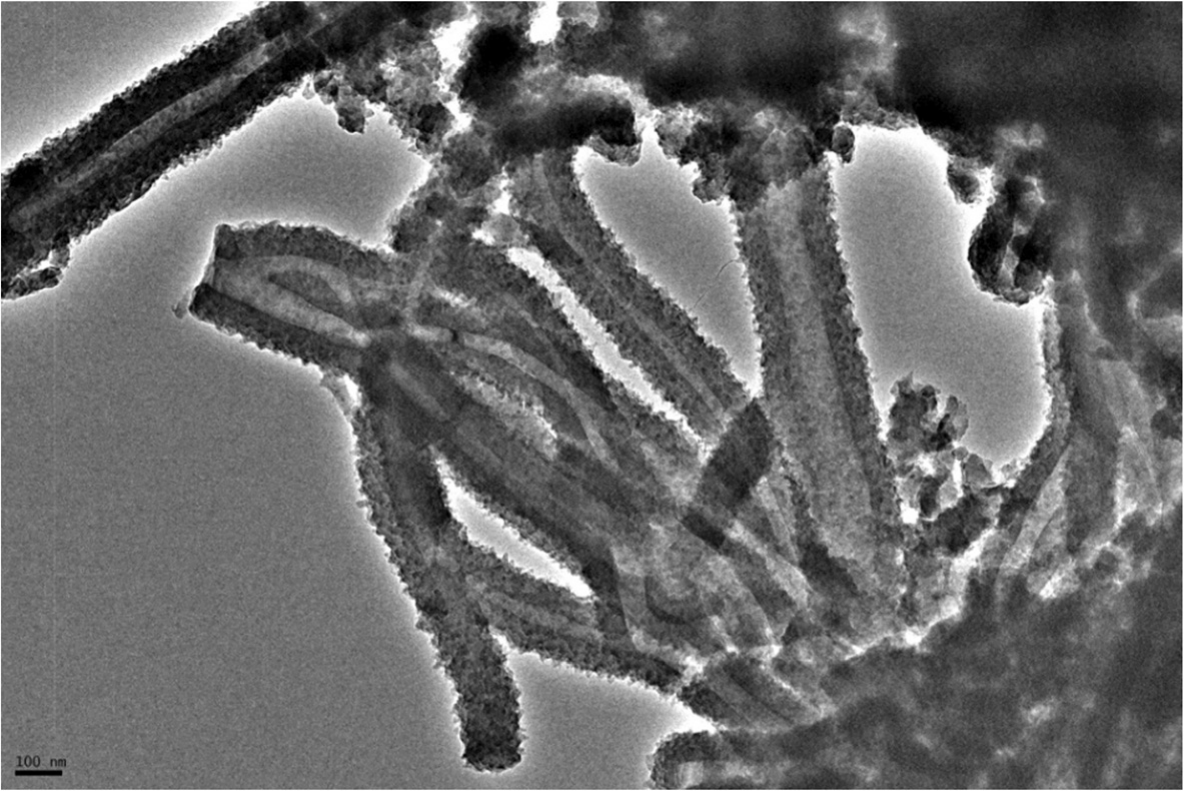
TEM of hollow nanotube zirconium tungesto-vanadate cation exchange material

Figures 3 and 4 identify the morphological structure of the microwave prepared zirconium tungesto-vanadate. SEM photographs clearly reveal that the material was produced at unique open-end tube architecture with average aspect ratio equal to 10. The measured outer and inner diameter of the produced material was around 30 and 7 nm, respectively. This hollow nanotube structure was confirmed with TEM image. Figure 4 investigated the homogenous hollow structure of the prepared zirconium tungesto-vanadate. This result indicated that the microwave technique in the presence of high molecular weight PVA as a stabilizing agent has the ability to architecture zirconium tungesto-vanadate at hollow nanotube morphological structure. This hollow structure nano-material is characterized by its high surface area that recorded as 640 m^2^/g. These results give prediction about the high reactivity of the prepared nano-material as cation exchanger [13].

### Assessment of Prepared Nanotube Zirconium Tungesto-Vanadate as Cation Exchange Material

**Fig. 5:**
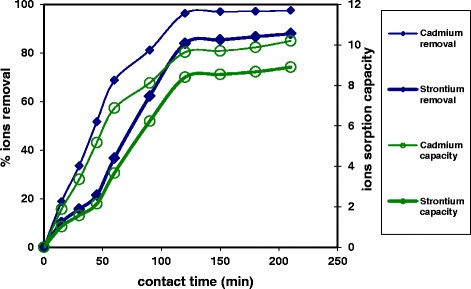
Sorption profiles of cadmium and strontium ions onto nanotube zirconium tungesto-vanadate cation exchange material

In order to confirm the ion exchange efficiency of the nanotube-produced material for cationic ions decontamination, the material sorption performance for cadmium and strontium ions were compared. Figure 5 investigated the high sorption affinity of the prepared material for the two studied cations, where the equilibrium state of the ion exchange processes of the two studied positive ions was achieved within 90 min. However, the material poses higher equilibrium sorption capacity of 9.88 mg/g for cadmium ions compared with 8.67 mg/g for strontium ions. This may be regarded to the smallest ionic hydrated radii of the cadmium ion that equal to 0.092 nm compared with the strontium ion ionic radii that is equivalent to 0.412 nm [14, 15]. Moreover, the percentage cadmium ion decontamination was recorded as 96.4 % within 90 min using the prepared hollow nanotube zirconium tungesto-vanadate material. This percentage of heavy metal ion decontamination is much higher compared with that recorded using the previously prepared zirconium tungesto-vanadate material that is produced in nanoparticle structure using sol-gel technique [6], where the nanoparticle zirconium tungesto-vanadate poses 96 % lead ion decontamination from the polluted wastewater within 3 h.

## Conclusions

A unique zirconium tungesto-vanadate cation exchange material was architectured at the hollow nanotube morphological structure using microwave technique in the presence of polyvinyl alcohol as a stabilizing agent. The material XRD examination investigated its pure crystalline structure. Its high cation exchange capacity value of 4.8 meq/g was confirmed through its affinity for cadmium and strontium cations. The prepared material accomplished higher cadmium sorption capacity of 9.88 mg/g for cadmium ions compared with 8.67 mg/g for strontium ions within 90 min. The innovative prepared material was presented as good nanotube cation exchanger for both heavy metals and radionuclides decontamination from polluted wastewaters.

## Abbreviations

BET: Brunauer–Emmett–Teller for surface area
ICP: plasma mass spectrophotometer
IEC: ion exchange capacity
LLRLW: low-level radioactive liquid waste
PVA: polyvinyl alcohol
SEM: scanning electron microscope
XRD: x-ray diffraction
